# Evaluating stability and bioactivity of Rehmannia-derived nanovesicles during storage

**DOI:** 10.1038/s41598-024-70334-5

**Published:** 2024-08-28

**Authors:** Xiaohang Chen, Lianghang He, Yao Chen, Genggeng Zheng, Yating Su, Yingcong Chen, Dali Zheng, Youguang Lu

**Affiliations:** 1 Fujian Key Laboratory of Oral Diseases, School and Hospital of Stomatology, Fujian Medical University, Fuzhou, China; 2 Department of Preventive Dentistry, School and Hospital of Stomatology, Fujian Medical University, Fuzhou, China

**Keywords:** Plant derived nanovesicles, Rehmannia, Stability, Bioactivity, Storage, Biotechnology, Cancer, Drug discovery, Plant sciences

## Abstract

Plant-derived nanovesicles (PDNVs) have garnered growing attention in the biomedical field owing to their abundance in plant-derived ribonucleic acids (RNA), proteins, lipids and metabolites. The question about the preservation of PDNVs is a crucial and unavoidable concern in both experiments’ settings and their potential clinical application. The objective of this research was to examine the impact of varying storage temperatures on the stability and bioactivity of Rehmannia-derived nanovesicles (RDNVs). The results showed that RDNVs aggregated after 2 weeks of storage period at 4 °C, and the particle size of some RDNVs gradually increased with time, along with the increase of solution potential. After 2 months of storage, all RDNVs exhibited varying levels of aggregation irrespective of storage temperature. The bioactivities of nanovesicles under different temperature storage conditions revealed a gradual decline in cell proliferation inhibition bioactivity over time, significantly lower than that of freshly prepared RDNVs. In contrast, the preservation of anti-migratory activity in RDNVs was found to be more effective when subjected to rapid freezing in liquid nitrogen followed by storage at − 80 °C, as opposed to direct storage at − 80 °C. These findings suggest that temperature alone may not be sufficient in safeguarding the activity and stability of RDNVs, highlighting the necessity for the development of novel protective agents for PDNVs.

## Introduction

Plant-derived nanovesicles (PDNVs) are lipid bilayer structures enriched with RNA, proteins, lipids and metabolic compounds from plants^1^. These rich components endow PDNVs with a variety of activities, such as antimicrobial, antioxidant, and immunomodulatory activities, which have promising applications in the fields of anti-tumor, tissue protection and repair^2,3^. Meanwhile, many PDNVs have been demonstrated to be well biocompatible, easily taken up by cells, and are green and renewable resources^4^. Despite many surprising discoveries, there is still no consensus on the preservation of PDNVs. This is an important issue because of the difficulties in obtaining fresh PDNVs in real time during experimental conduct and clinical application, e.g., fresh flowers, fruits, and rhizomes of certain plants are only present in certain seasons.

Given the research on animal-derived extracellular vesicles, storage at 4 °C is recommended for short-term use, while placement at − 80 °C is recommended for long-term storage^5^. In current studies, researchers usually store freshly obtained PDNVs directly at − 80 °C^6,7^, a few studies store them at − 20 °C^8^, and a very few studies store them at − 80 °C after liquid nitrogen flash freezing^9,10^. Meanwhile, studies have been conducted to evaluate the stability of blueberry and dendrobium leaf-derived nanovesicles under different storage conditions, and it was found that different storage conditions affect the protein content, surface charge, cellular uptake efficiency, and particle size distribution of PDNVs^11,12^. However, the preservation conditions of nanovesicles from different plants should not be generalized due to the differences in nanovesicles from different plants.

Rehmannia is a commonly used herb in traditional Chinese medicine^13^. The pharmacological effects of Rehmannia are mainly attributed to its various active components, including flavonoids, tanshinones, flavonoid glycosides, phenylethanol glycosides and amino acids^14^. These active constituents endow Rehmannia with bioactivities such as immunomodulation, antioxidant and hepatoprotective activities, which have promising applications in the fields of tissue protection and antitumour^15,16^. The pharmacological value of Rehmannia-derived nanovesicles (RDNVs) have been previously studied. Qiu et al. isolated RDNVs and found that they could ameliorate lipopolysaccharide-induced acute lung injury and intestinal flora dysbiosis^17^. In addition, our unpublished study showed that RDNVs could be taken up by cells and had activities that inhibited the proliferation and migration of squamous cell carcinoma cells. However, there have been no studies on how to preserve RDNVs, which poses challenges such as batch stability of experiments.

Therefore, the objective of this study was to investigate the stability and bioactivity of RDNVs under different storage temperatures to find a suitable preservation condition. We evaluated the morphological changes, potential alterations and particle size distribution of the RDNVs at 4 °C, − 20 °C, − 80 °C and liquid nitrogen quick-freezing followed by transfer to − 80 °C storage. More importantly, we evaluated the changes in bioactivity of RDNVs at different storage temperatures to determine the optimal preservation conditions. This will draw attention to the storage of PDNVs.

## Methods and materials

### Cell culture

LN4 cells, an oral squamous cell carcinoma cell line with high lymph node metastatic properties constructed by the group in the previous stage, had been identified by short tandem repeat (STR) ^18^. Cells were cultured at 37 °C and 5% CO_2_ until the growth confluence reached 80%, then digested and passaged using trypsin(Gibco, USA).

### Isolation of RDNVs

We actively washed fresh rehmannia (Jiaozuo, China) with running water, followed by three additional washes with distilled water. Subsequently, we chopped it and juiced it with cold phosphate-buffered saline (PBS) using a juicer. After the juicing process with a ratio of 1 g of rehmannia to 2 mL of PBS, we filtered out the residue and subjected the remaining liquid to low-speed centrifugation. We centrifuged it first at 2000 g for 10 min, followed by 5000 g for 20 min, and finally 10,000 g for 1 h to remove debris and residue, respectively. To prevent the difficult resuspension caused by nanovesicle aggregation, we used centrifugation at 100,000 g for 30 min to collect nanovesicles^19^. After that, we subjected the obtained nanovesicles to two additional washes using 100,000 g for 30 min to eliminate impurities from the solution. After decontamination using a 0.22 μm filter membrane, we utilized them for downstream experiments.

### Bicinchoninic acid (BCA) protein quantification of RDNVs

For BCA protein quantification of RDNVs, different volumes (0, 1, 2, 4, 8, 12, 16, 20 μL) of the same concentration (0.5 mg/mL) of bovine serum albumin (BSA) were used to formulate a 20 μL reaction system with PBS, respectively. Sample wells to be tested were added with 2 μL of sample and 18 μL of PBS. Then, 200 μL of 50:1 configuration of BCA reagent (Beyotime, Shanghai) solution of solution A and solution B were added to each system. After incubation at 37 °C for 30 min, the absorbance (OD) values were detected at 562 nm using an microplate reader (SpectraMax iD3). A standard curve was plotted based on the values obtained and substituted to calculate the concentration of the samples.

### Storage of RDNVs

The nanovesicles quantified by BCA were diluted to 1 mg/mL with PBS at 4 °C and divided into (1) liquid nitrogen quick-freezing and then transferred to − 80 °C group, (2) direct storage at − 80 °C group, (3) direct storage at − 20 °C group, and (4) direct storage at 4 °C group. To prevent repeated freezing and thawing, each sterile freezing tube was labelled in advance as to its purpose and storage time, and was only removed when used for bioactivity assessment and testing.

### Potential and particle size distribution assessment of RDNVs

Prior to potential and particle size testing, frozen RDNVs were thawed rapidly in a 37 °C water bath and diluted for measurement immediately after the samples were thawed and fully resuspension. 100 μL of 1 mg/mL RDNVs was taken and diluted using 900 μL of PBS liquid and the potential and particle size distribution of the solutions were tested using a dynamic light scatterometry (Anton Paar, Austria) under the same conditions using quartz potentiostat dishes and normal PVC dishes respectively.

### Morphological assessment of RDNVs

RDNVs were observed under transmission electron microscope (FEI TecnaiG2, Japan). Firstly, after the solution was fully resuspended, 5 μL of RDNVs was taken on a clean PVC membrane and mixed thoroughly with 5 μL of 4% paraformaldehyde. Then, a 400-mesh copper mesh was covered and fixed for 30 min. Subsequently, the mixed liquid was removed and washed three times with distilled water. The residual liquid was blotted out using filter paper and stained with 2% phosphotungstic acid solution (pH = 7) for 2 min. The staining liquid was removed and washed 3 times with distilled water. Finally, the residual liquid was blotted out using filter paper and dried at room temperature for more than 30 min for observation.

### Cell viability assay

Cells were seeded in 96-well plates at a density of 2000 cells per well. The solution was configured into four concentrations of 50 μg/mL, 25 μg/mL, 12.5 μg/mL and 6.25 μg/mL using complete medium. The intervention was carried out by adding the appropriate concentration of drug at 24 h after cell implantation. At 48 h after addition of RDNVs, the medium was aspirated and CCK8 solution (Invitrogen) diluted in a 1:9 ratio was added. A complete blank group was set up and 100 μL of liquid was added to each well and incubated for 1 h. The absorbance (OD) values were measured at 450 nm using an microplate reader(SpectraMax iD3).

Cell viability (%) = ((OD experimental group—OD complete blank group) / (OD blank control group—OD complete blank group)) × 100%

### Scratch assay

Cells were grown in 12-well plates with 800,000 cells per well. After the cells had grown to approximately 90%, a “cross” scratch was created on the cell monolayer using a 200 μL pipette tip. Post-scratch interventions were performed using serum-free medium configured with 50 μg/mL RDNVs. Photographs were taken at 0 h at the beginning of the scratch intervention and again when the scratch healed in the blank control group (approximately 30 h). The images were captured at the upper and lower intersections of the “cross” scratch. The blank area was analyzed using ImageJ software.

Scratch healing rate (%) = ((initial area—end point area)/initial area) × 100%

### Statistical analysis

Numerical results were expressed as mean ± standard deviation. Comparisons between groups were made using the appropriate t-test for comparing numerical data between two groups, while ANOVA was applied to compare three or more groups. Multiple comparisons were used to determine significant differences between specific groups when significant differences existed. The level of significance was set at *p* < 0.05.

### Statement

The plant material, Rehmannia, used in this study has been identified and authenticated. It was purchased from Wen County, Jiaozuo, Henan Province, China, and originated from a farm where it was legally cultivated and harvested for commercial purposes. Experimental research and field studies on plants (either cultivated or wild), including the collection of plant material, were carried out in compliance with relevant institutional, national, and international guidelines and legislation.

The LN4 cells line was derived from the oral squamous cell carcinoma Cal-27 cell line, which was purchased from ATCC. The detailed construction process is described in our previous work^18^. The LN4 cells line exhibits enhanced lymph node metastasis capabilities, with STR analysis confirming the retention of Cal-27 cell fingerprinting information.

## Results

### Effect of different preservation temperatures on the morphology of RDNVs

**Figure 1:**
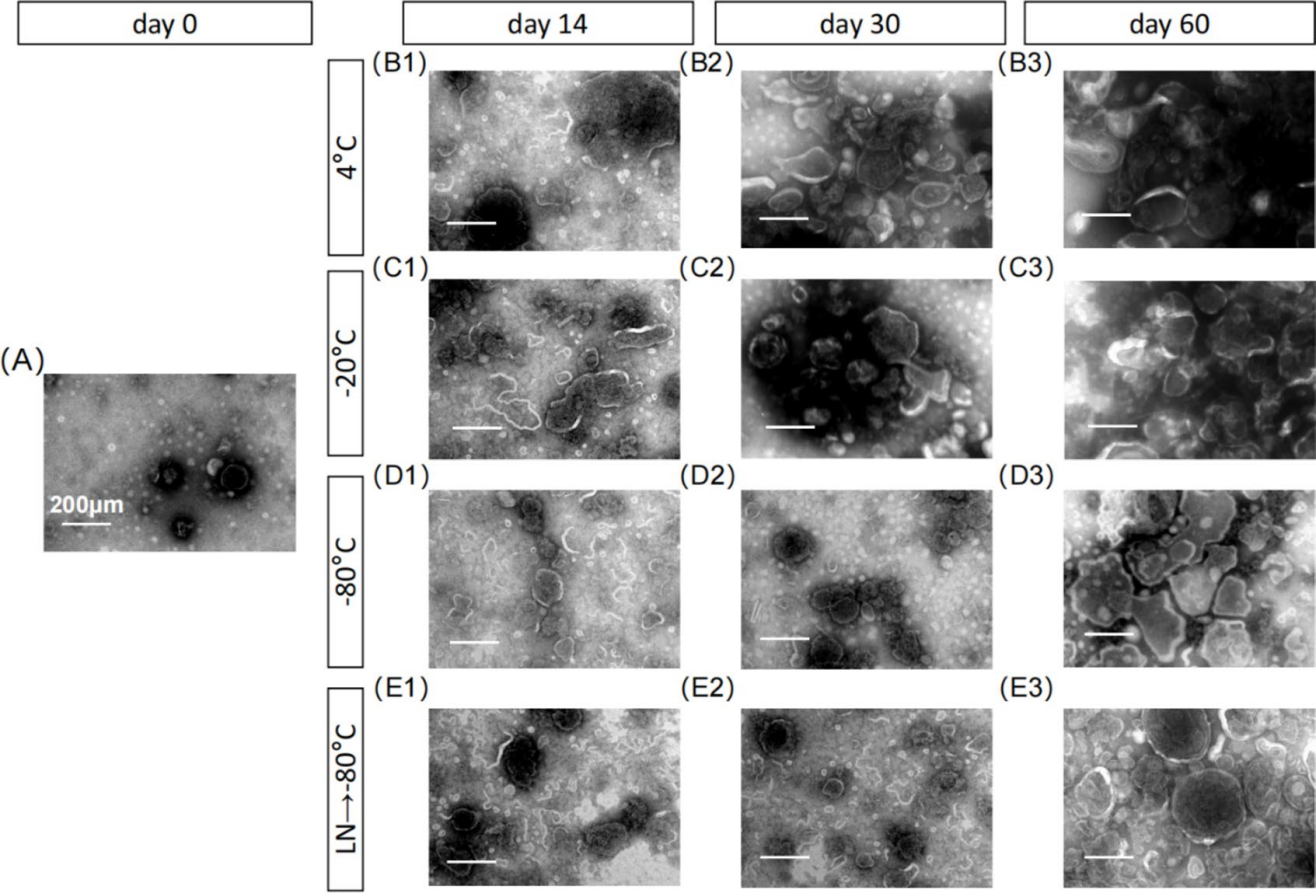
Electron microscopic morphology of RDNVs under different storage temperature and storage time conditions. (**A**) The electron microscopy images of fresh RDNVs. (**B1**–**B3**) The electron microscopy images of RDNVs under 4 °C storage conditions at day 14, day 30, and day 60. (**C1–C3**) The electron microscopy images of RDNVs under − 20 °C storage conditions at day 14, day 30, and day 60. (**D1–D3**) The electron microscopy images of RDNVs under − 80 °C storage conditions at day 14, day 30, and day 60. (**E1–E3**) The electron microscopy images of RDNVs under − 80 °C following rapid freezing in liquid nitrogen storage conditions at day 14, day 30, and day 60. (RDNVs, Rehmannia derived nanovesicles; LN, liquid nitrogen.)

We investigated the effects of different preservation conditions on the physicochemical characteristics of RDNVs. Firstly, we visualized the morphology of RDNVs. The results showed that at the beginning, the vesicles were more dispersed(Fig. 1A). However, after 2 weeks of storage at 4 °C, a significant fusion aggregation of RDNVs was observed (Fig. 1B1–B3). We also observed the occurrence of aggregation and fusion under the − 20 °C storage conditions (Fig. 1C1–C3). However, the effects were less pronounced under the − 80 °C and after transferring to − 80 °C following rapid freezing in liquid nitrogen (Fig. 1D1–D3, E1–E3). And the degree of fusion gradually increased. After one month, the RDNVs showed significant aggregation under the storage conditions of 4 °C and − 20 °C (Fig. 1B2, C2), while the dispersion remained relatively unaffected under the − 80 °C storage conditions and after transferring to − 80 °C following rapid freezing in liquid nitrogen (Fig. 1D2, E2). However, at 2 months, we observed aggregation and fusion of RDNVs regardless of the storage temperature (Fig. 1B3, C3, D3, E3).

### Effect of different preservation temperatures on the particle size distribution of RDNVs

**Figure 2:**
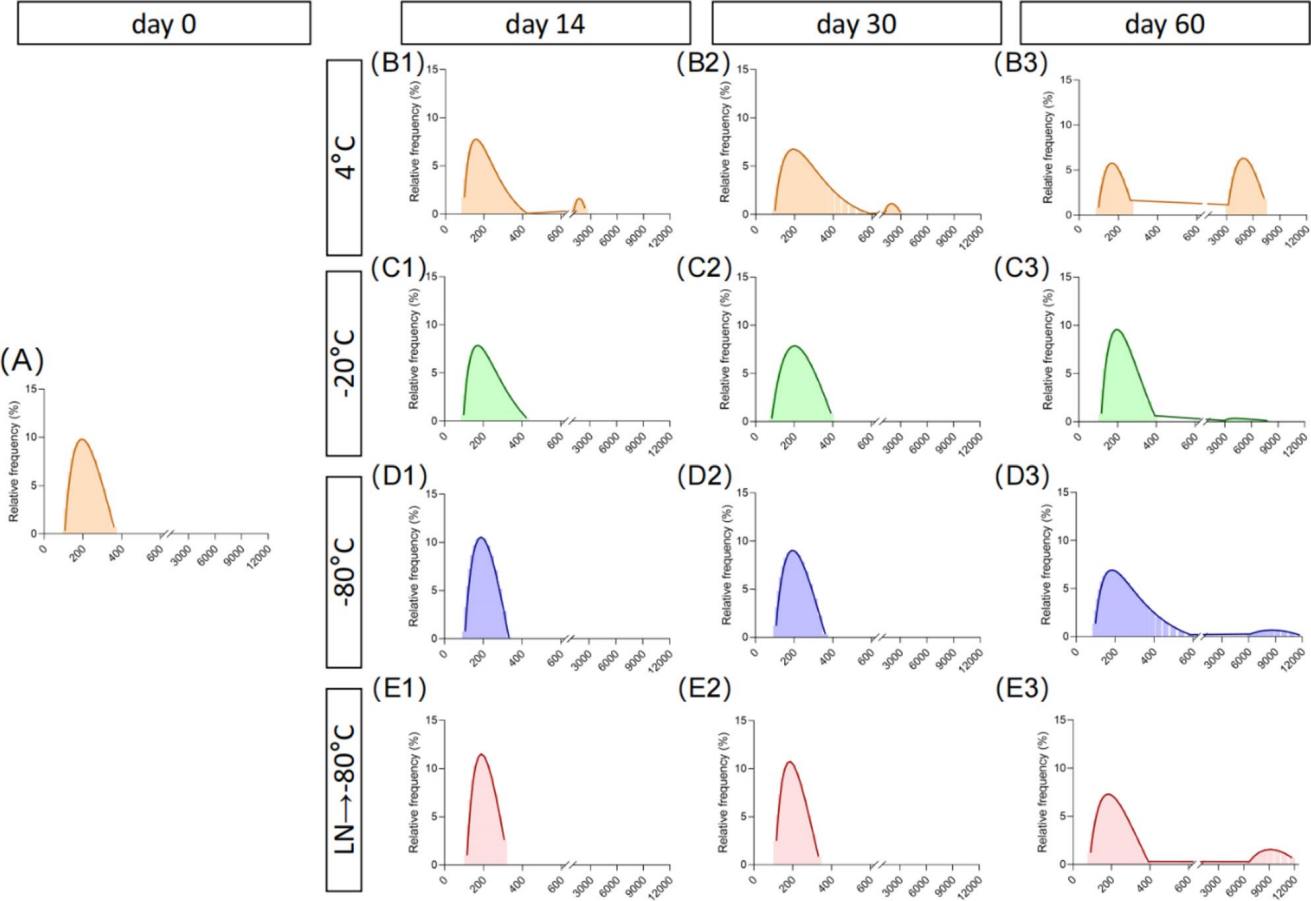
Particle size distributions of RDNVs under different preservation temperature conditions and preservation time. (**A**) The particle size distributions of fresh RDNVs. (**B1–B3**) The particle size distributions of RDNVs under 4 °C storage conditions at day 14, day 30, and day 60. (**C1–C3**) The particle size distributions of RDNVs under − 20 °C storage conditions at day 14, day 30, and day 60. (**D1–D3**) The particle size distributions of RDNVs under − 80 °C storage conditions at day 14, day 30, and day 60. (**E1–E3**) The particle size distributions of RDNVs under − 80 °C following rapid freezing in liquid nitrogen storage conditions at day 14, day 30, and day 60. (RDNVs, Rehmannia derived nanovesicles; LN, liquid nitrogen.)

Subsequently, we evaluated the dynamic distribution of the particle size of RDNVs under different preservation temperature conditions using dynamic light scattering. We observed that the average particle size of the RDNVs was about 200 nm initially (Fig. 2A), and after 2 weeks of storage at 4 °C, some of the RDNVs had undergone aggregation or fusion (Fig. 2B1). At 30 days, the size distribution peak of the vesicles under the 4 °C storage condition showed a tendency towards lower dispersion, indicating a more pronounced increase in size (Fig. 2B2). After storage at − 20 °C for 14 and 30 days, there was an increasing trend in the size of RDNVs (Fig. 2C1, 2). However, the size of RDNVs stored at − 80 °C and after transferred to − 80 °C storage condition following flash-freezing in liquid nitrogen showed no significant changes in size within 1 month (Fig. 2D1–D2, and 2E1–E2). However, after two months of storage, all these vesicles started to show noticeable aggregation (Fig. 2D3 and E3), with the most severe aggregation observed under the 4 °C storage condition (Fig. 2B3). This result is consistent with the observations made under electron microscopy, where RDNVs also exhibited fusion and aggregation after storage.

### Effect of different preservation temperatures on the potential distribution of RDNVs

**Figure 3:**
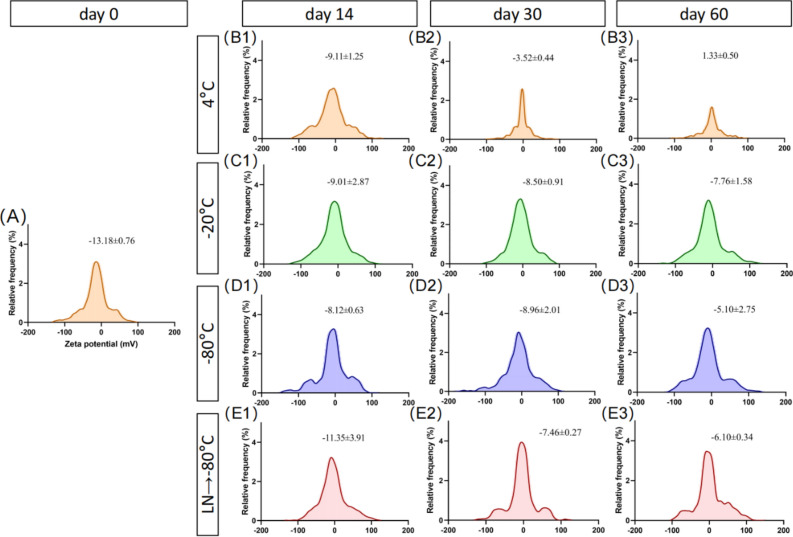
Potential distributions of RDNVs under different preservation temperature and preservation time conditions. (**A**) The particle potential distributions of fresh RDNVs. (**B1–B3**) The particle potential distributions of RDNVs under 4 °C storage conditions at day 14, day 30, and day 60. (**C1–C3**) The particle potential distributions of RDNVs under − 20 °C storage conditions at day 14, day 30, and day 60. (**D1–D3**) The particle potential distributions of RDNVs under − 80 °C storage conditions at day 14, day 30, and day 60. (**E1–E3**) The particle potential distributions of RDNVs under − 80 °C following rapid freezing in liquid nitrogen storage conditions at day 14, day 30, and day 60. (RDNVs, Rehmannia derived nanovesicles; LN, liquid nitrogen.)

The negative charges on the surface of the lipid bilayer may mutually repel each other, which could be a key factor in maintaining the stability of the solution^20^. However, due to the presence of not only lipids but also proteins and other components in this vesicular structure^19^, long-term storage may affect the changes in the solution potential of RDNVs. As such, we further evaluated the dynamic potential distribution of the RDNVs under different preservation temperature conditions. We observed that initially the potential of the RDNVs was around − 12 mV (Fig. 3A). At the end of 2 weeks of storage, the zeta potentials of RDNVs solutions in all subgroups increased (Fig. 3B1, C1, D1), except for that were transferred to − 80 °C after liquid nitrogen rapid freezing group, which did not show significant changes (Fig. 3E1). When evaluated at the next 1- and 2-month time points, particle potentials under 4 °C storage conditions changed significantly from negative to positive potentials, with a potential increase of about 14 mV (Fig. 3B1–B3). Overall, the potential distribution of RDNVs under − 20 °C storage conditions was more stable, with only a slight upregulation of approximately 4 mV after two months of storage (Fig. 3C1–C3). Compared to transferring to − 80 °C after liquid nitrogen flash-freezing, RDNVs stored at − 80 °C showed an earlier upregulation of potential (Fig. 3D1–D3). However, there was no significant difference in potential between RDNVs stored for 1–2 months after being transferred to − 80 °C after liquid nitrogen rapid freezing and those directly stored at − 80 °C (Fig. 3E1–E3).

### Effect of different preservation temperatures on the ability of RDNVs to inhibit tumor cell proliferation

**Figure 4:**
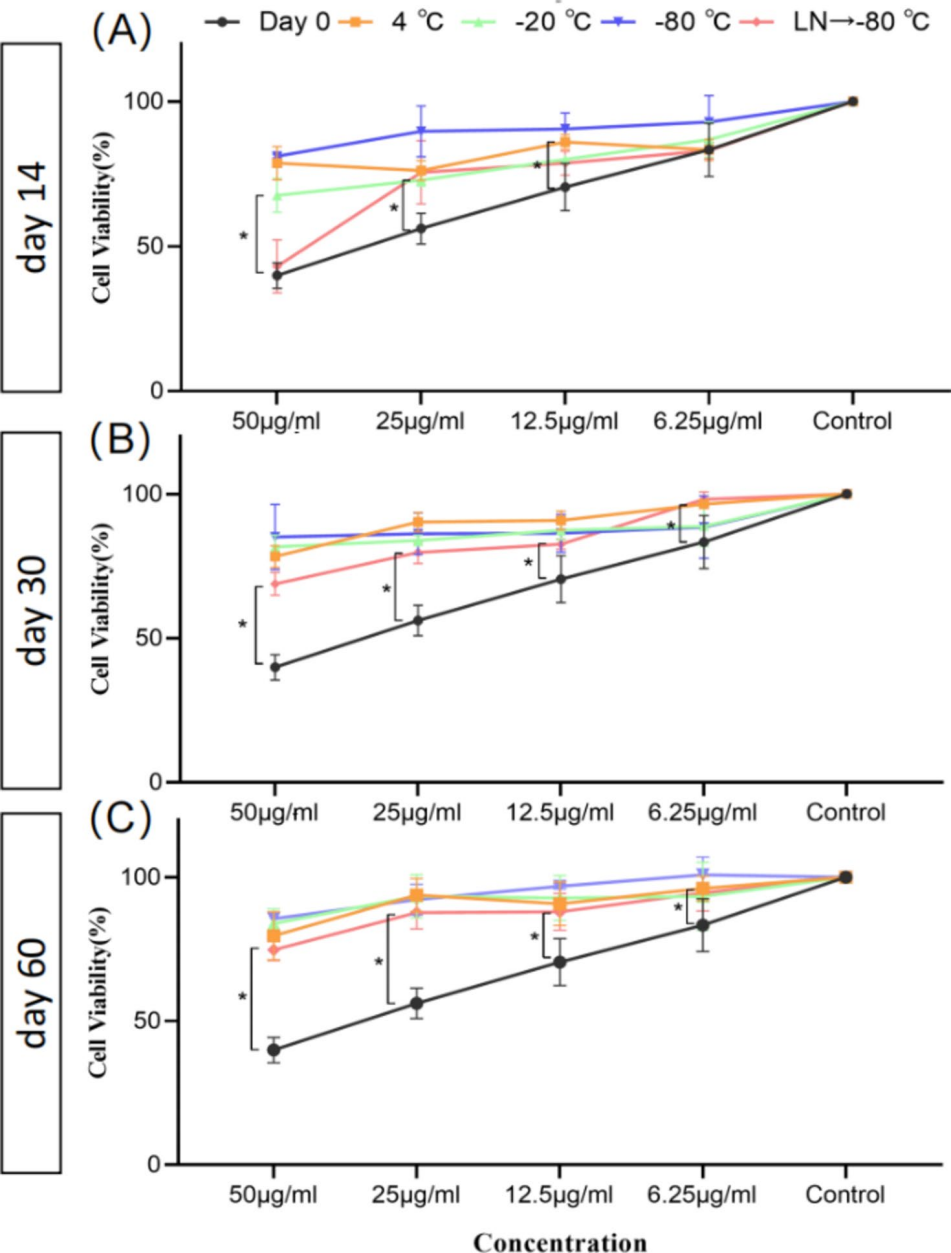
Temporal changes in the inhibitory effect of RDNVs on cell proliferation under different temperature storage conditions. (**A**) Changes in the inhibitory effect of RDNVs on cell proliferation at different concentrations after 14 days of storage at different temperatures. (**B**) Changes in the inhibitory effect of RDNVs on cell proliferation at different concentrations after 30 days of storage at different temperatures. (**C**) Changes in the inhibitory effect of RDNVs on cell proliferation at different concentrations after 60 days of storage at different temperatures.

Currently, there have been studies characterizing the physical properties of PDNVs under different temperature storage conditions^11,12^. However, there has been a lack of systematic tracking and evaluation of the bioactivity of these PDNVs. Subsequently, we evaluated the effect of RDNVs on LN4 cell viability under different preservation temperature conditions. At the initial isolated nanovesicles at a concentration of 50 μg/mL, 50% of cell viability could be inhibited after 48 h of action, while some inhibitory effect on LN4 cell viability was also observed at a concentration of 6.25 μg/mL (Fig. 4A). Under the storage condition of 4 °C, except for RDNVs with a concentration of 50 μg/mL, which maintained their proliferative activity in LN4 cells after 60 days of storage, all other concentrations became ineffective (Fig. 4C). Under the storage condition of − 20 °C, RDNVs could still inhibit the proliferation activity of LN4 cells to varying degrees after 30 days of storage, but the efficacy decreased compared to freshly RDNVs (Fig. 4B). Surprisingly, storing RDNVs directly at − 80 °C for 14 days significantly reduced their anti-proliferative activity (Fig. 4A). On the other hand, when RDNVs were rapid freezing in liquid nitrogen and then transferred to − 80 °C, they showed better preservation of RDNVs' bioactivity compared to other conditions (Fig. 4A–C). However, none of these conditions could ideally preserve the anti-proliferative activity of RDNVs.

### Effect of different preservation temperatures on the ability of RDNVs to inhibit tumor cell migration

**Figure 5:**
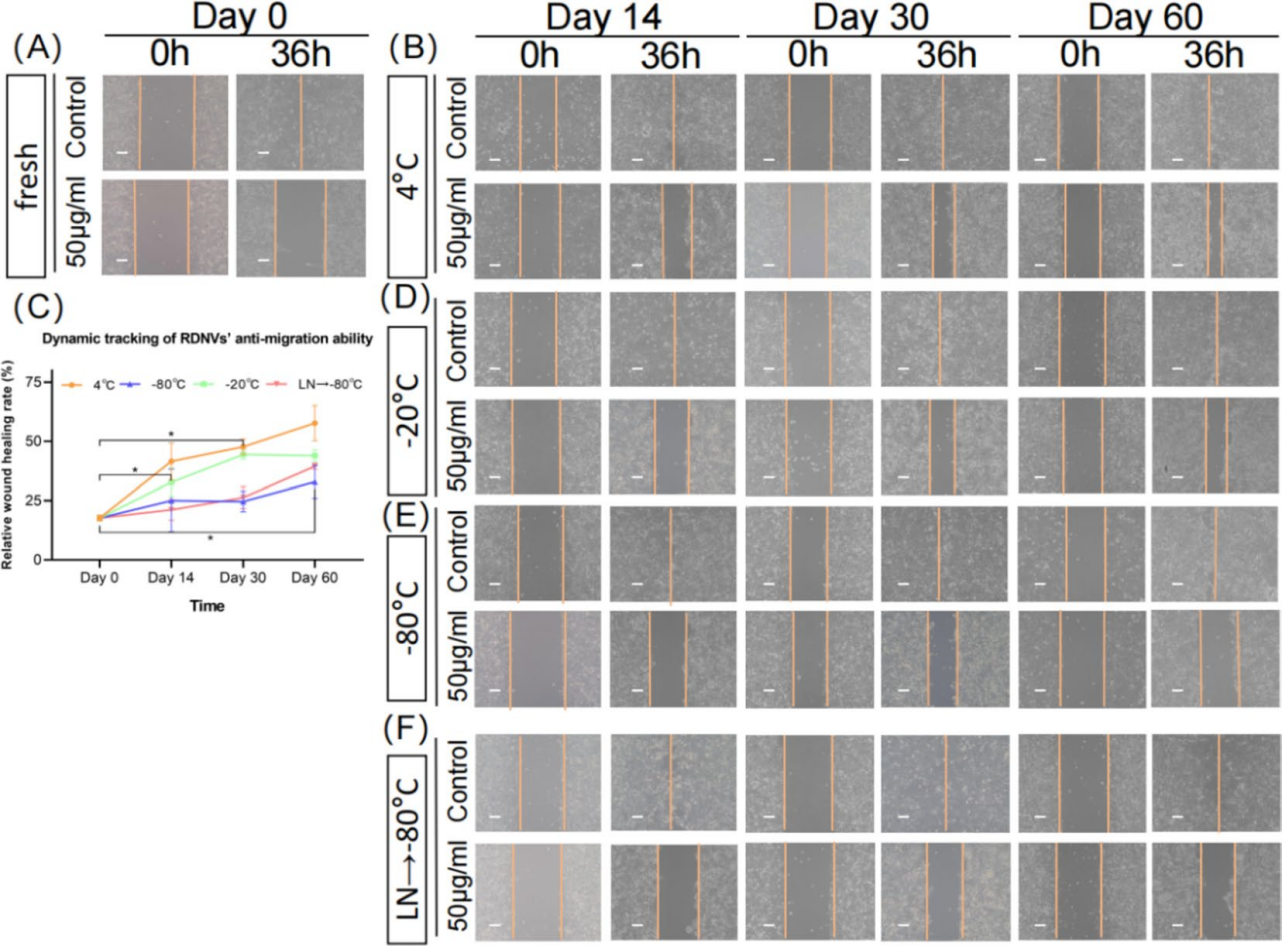
Dynamic changes in the effects of RDNVs on the motility of LN4 cells under different storage temperatures. (**A**) Effects of fresh RDNVs on the motility of LN4 cells. (**B**) Effects of RDNVs on cell motility after storage at 4 °C for 14, 30, and 60 days. (**C**) Relative inhibition of LN4 cell movement by RDNVs compared to the control group after storage at different temperatures for different durations. (**D**) Effects of RDNVs on cell motility after storage at − 20 °C for 14, 30, and 60 days. (**E**) Effects of RDNVs on cell motility after storage at − 80 °C for 14, 30, and 60 days. (**F**) Effects of RDNVs on cell motility after storage at − 80 °C following rapid freezing in liquid nitrogen for 14, 30, and 60 days.

Furthermore, we also assessed the impact of different storage conditions on the ability of RDNVs to inhibit LN4 cell migration. We observed that fresh RDNVs at a concentration of 50 μg/mL could reduce cell motility by approximately 75%. However, under 4 °C storage conditions, the ability to inhibit cell movement decreased by almost half after 60 days (Fig. 5A–C). Under − 20 °C storage conditions, the ability of RDNVs to inhibit LN4 cell migration decreased over time (Fig. 5A, C, and D). Within a storage time of 14 days, RDNVs maintained their bioactivity in  rapid freezing in liquid and transferred to − 80 °C storage conditions, performing at levels comparable to fresh samples. After 2 months of storage, the bioactivity of the vesicles remained present under − 80 °C storage and liquid nitrogen snap-freezing followed by transfer to − 80 °C storage conditions, albeit with a slight reduction in RDNVs bioactivity (Fig. 5A, C, E, and F).

## Discussion

PDNVs have shown various advantages in the field of disease treatment, such as good biocompatibility, low toxicity, drug loading capacity, and easy modifiability^4,21^. They have shown great potential in areas such as tissue regeneration and anti-tumor effects, and are expected to inject new power into the development of cosmetics and drugs^1,22^. Rehmannia is a commonly used plant with extensive medicinal value, and it has been shown to have potential in areas such as cognitive improvement^23^, anti-tumor effects^24^, and tissue protection^25^. Rehmannia-derived nanovesicles have been found can mitigate acute lung injury induced by lipopolysaccharides and restore intestinal microbiota balance^17^. Our previous research discovered the potential of RDNVs in inhibiting squamous cell carcinoma, but this bioactivity varies among nanovesicles derived from Rehmannia purchased in the same region but during different seasons. This suggests the need for proper preservation of RDNVs and also poses a challenge for batch stability of RDNVs. Common methods for preserving PDNVs include low-temperature storage (such as freezing or refrigeration), freeze-drying method, and the addition of protective agents. Most of the literature uses − 80 °C as a storage condition for plant-derived nanovesicles. Other articles have used rapid freezing with liquid nitrogen or rapid freezing with liquid nitrogen followed by freeze-drying to preserve PDNVs, as rapid freezing with liquid nitrogen is expected to reduce ice crystal formation^9^. Leng et al. evaluated the stability of blueberry-derived nanovesicles stored at different temperatures (including 4 °C, − 20 °C, and − 80 °C) and found that short-term storage at 4 °C and long-term storage at − 80 °C better maintained the stability of the vesicles, although the study tracked them for 30 days^12^. In our study, we extended the time to 2 months initially and found that prolonged preservation by cryopreservation may not be good enough to maintain nanovesicles stability.

In this study, we tracked the changes in particle size, morphology, and potential of RDNVs at different storage temperatures and over different time periods. We found that regardless of the storage temperature, RDNVs experienced reduced potential and phenomena such as fusion and aggregation after approximately 2 months of storage. This is similar to conclusions drawn from previous studies. In this study, we introduced a group that underwent rapid freezing with liquid nitrogen followed by storage at − 80 °C, which is a storage condition used in some studies. We found that this storage condition appeared to better maintain vesicle stability in the short term, suggesting that direct rapid freezing with liquid nitrogen and continuous storage in liquid nitrogen may yield better results. However, it seems difficult to reach a definitive conclusion about which temperature is more suitable for storing a particular type of vesicle to maintain its stability for a long time. In Zeng et al.’s study, it was found that nanovesicles derived from aloe vera could be well maintained at − 20 °C, and they speculated that this may be due to the presence of certain components in the nanovesicles from aloe vera that have better stabilizing effects^19^. On the other hand, in Ge et al.’s study, they found that nanovesicles derived from Brucea javanica did not show significant changes in size and particle size after one year of storage at − 80 °C^26^. These studies suggest that there may be inherent differences in the stability of PDNVs themselves. The storage conditions of nanovesicles from different plant sources may vary. When studying the storage conditions of nanovesicles, the effects on vesicle activity and stability should be considered simultaneously. On the one hand, the storage conditions should maintain the structural integrity and functional activity of the nanovesicles to ensure the effective release of the active ingredients at the time of use. On the other hand, the storage conditions should prevent irreversible changes in the composition and properties of the nanovesicles and maintain their consistency and reproducibility.

In this study, we also evaluated the anti-proliferative and anti-migratory activities of RDNVs against oral squamous cell carcinoma cells at different storage temperatures. We found that, apart from the storage condition of liquid nitrogen snap-freezing followed by transfer to − 80 °C, which preserved the anti-proliferative activity of RDNVs for 2 weeks, other temperature conditions failed to maintain the anti-proliferative activity of RDNVs. However, the anti-migratory ability was not significantly compromised as the anti-proliferative activity. Storage conditions at -20 °C, − 80 °C, and rapid freezing with liquid nitrogen followed by storage at − 80 °C all maintained good activity at 2 months, with the best results observed for the latter condition. This differential result may be attributed to the fact that RDNVs are nanovesicles containing multiple components, and different active ingredients in the vesicles may contribute to their anti-proliferative and migration-inhibiting effects.

Also, this difference in bioactivity may be due to the fact that after fusion and aggregation of PDNVs, their morphology and stability can influence the uptake pathway by which they enter the cell. For example, nanoparticles with a size of 150–200 nm are mainly taken up by endocytosis mediated by clathrin or caveolin mediated endocytosis, whereas nanovesicles with a size of 250 nm–3 μm are taken up by cells in the form of macropinocytosis and phagocytosis^27^. The different uptake efficiencies would further lead to changes in the bioavailability of the contents of the RDNVs, which in turn would affect the biological activities they exerts.

Therefore, future research can explore more precise and controllable storage conditions^5^. This includes optimizing parameters such as storage temperature, humidity, as well as introducing new protective agents or preservatives^28^. These protective agents can safeguard the structure and functionality of nanovesicles during storage and mitigate adverse effects from the external environment. They provide additional protection to maintain the stability and activity of nanovesicles. Common protective agents include sucrose, protein stabilizers, and antioxidants^29^. When researching protective agents for PDNVs, the impact of the agents on the nanovesicles and their compatibility with them must be considered. The protective agents should interact with the nanovesicles to preserve their structural and functional integrity, prevent aggregation or degradation during storage. Additionally, the protective agents should exhibit good biocompatibility to ensure no adverse effects on human or biological environments upon application. In recent years, studies have evaluated the effectiveness of protective agents in preserving the stability of PDNVs. Kim et al. assessed the effectiveness of 1,3-butanediol or TMO in protecting the stability of nanovesicles derived from Dendrobium leaves^11^. They set four temperatures (− 20 °C, 4 °C, 25 °C, and 45 °C) and stored the nanovesicles for four weeks, comparing the stability of fresh nanovesicles with those protected by the agents. They found that TMO provided better protection for nanovesicles derived from Dendrobium leaves when stored at 4 °C compared to 1,3-butanediol. This suggests that protective agents may serve to safeguard PDNVs. However, the study only evaluated stability and did not address whether the protective agents have any additional impact on the activity of PDNVs during long-term protection or whether they can maintain the sustained bioactivity of PDNVs.

Using some natural products as protective agents may be a promising direction^30^. For example, certain polysaccharides like trehalose have been found to improve the stability and preservation of cell-derived extracellular vesicles^31^. In a study on the isolation of nanovesicles from tobacco, it was found that the addition of trehalose reduced their aggregation during the isolation process. This raises the question of whether it could potentially enhance the stability of vesicles during the storage process, which is worth investigating^32^. Additionally, natural antioxidants such as vitamin C and glutathione hold potential for protecting nanovesicles. Furthermore, the effects of protective agents under different storage conditions should be investigated. For instance, the suitability of protective agents may differ between cryopreservation and lyophilization. Therefore, it is necessary to evaluate the effectiveness and compatibility of different protective agents under various storage conditions.

In future research, a systematic evaluation of the effectiveness of various protective agents in different applications of PDNVs and their interactions with different storage conditions can be conducted. This will help determine the optimal protective agents and storage methods to maintain the bioactivity and stability of PDNVs for better utilization in drug delivery and other biomedical applications.

## Conclusion

This study aims to investigate the effects of different storage temperatures on the stability and bioactivity of RDNVs. We found that regardless of the storage temperature, RDNVs undergo fusion aggregation and a decrease in potential after long-term preservation. Furthermore, we discovered that different storage temperatures have varying effects on the anti-proliferation and anti-migration activities of RDNVs. Conditions involving rapid freezing in liquid nitrogen followed by storage at − 80 °C showed better preservation of RDNV bioactivity in the short term. These results indicate that relying solely on low-temperature refrigeration may not be sufficient to effectively protect the activity and stability of RDNVs, necessitating the urgent development of new PDNVs protectants. These protectants should maintain the structure and function of vesicles during storage while exerting no additional impact on vesicles activity, thus better preserving vesicles activity and providing assurance for the future industrialization of PDNVs.

## Data Availability

Raw data can be requested from the corresponding author upon reasonable request.

## References

[1] Chen, X. *et al.* Plant-derived nanovesicles: Harnessing nature’s power for tissue protection and repair. *J. Nanobiotechnol.* **21**(1), 445 (2023). 10.1186/s12951-023-02193-7 PMC10668476 38001440

[2] Lian, M. Q. *et al.* Plant-derived extracellular vesicles: Recent advancements and current challenges on their use for biomedical applications. *J. Extracell. Vesicles* **11**(12), e12283 (2022). 36519808 10.1002/jev2.12283 PMC9753580

[3] Chen, X. *et al.* Engineered plant-derived nanovesicles facilitate tumor therapy: Natural bioactivity plus drug controlled release platform. *Int. J. Nanomed.* **18**, 4779–4804 (2023). 10.2147/IJN.S413831 PMC10460188 37635909

[4] Dad, H. A., Gu, T. W., Zhu, A. Q., Huang, L. Q. & Peng, L. H. Plant exosome-like nanovesicles: Emerging therapeutics and drug delivery nanoplatforms. *Mol. Ther. J. Am. Soc. Gene Ther.* **29**(1), 13–31 (2021). 10.1016/j.ymthe.2020.11.030 PMC7791080 33278566

[5] Görgens, A. *et al.* Identification of storage conditions stabilizing extracellular vesicles preparations. *J. Extracell. Vesicles* **11**(6), e12238 (2022). 35716060 10.1002/jev2.12238 PMC9206228

[6] Chen, Q. *et al.* Natural exosome-like nanovesicles from edible tea flowers suppress metastatic breast cancer via ROS generation and microbiota modulation. *Acta Pharm. Sin. B* **12**(2), 907–923 (2022). 35256954 10.1016/j.apsb.2021.08.016 PMC8897038

[7] Ou, X. *et al.* Novel plant-derived exosome-like nanovesicles from *Catharanthus roseus*: Preparation, characterization, and immunostimulatory effect via TNF-α/NF-κB/PU.1 axis. *J. Nanobiotechnol.* **21**(1), 160 (2023). 10.1186/s12951-023-01919-x PMC10199296 37210530

[8] Zeng, L. *et al.* Codelivery of π-π stacked dual anticancer drugs based on aloe-derived nanovesicles for breast cancer therapy. *ACS Appl. Mater. Interfaces* **14**(24), 27686–27702 (2022). 35675505 10.1021/acsami.2c06546

[9] Hwang, J. H. *et al.* Yam-derived exosome-like nanovesicles stimulate osteoblast formation and prevent osteoporosis in mice. *J. Control. Release* **355**, 184–198 (2023). 36736431 10.1016/j.jconrel.2023.01.071

[10] Kilasoniya, A., Garaeva, L., Shtam, T., Spitsyna, A., Putevich, E., Moreno-Chamba, B., Salazar-Bermeo, J., Komarova, E., Malek, A., Valero, M. & Saura, D. Potential of Plant Exosome Vesicles from Grapefruit (*Citrus* × *paradisi*) and Tomato (*Solanum lycopersicum*) Juices as Functional Ingredients and Targeted Drug Delivery Vehicles. *Antioxidants (Basel, Switzerland)*, **12**(4) (2023). 10.3390/antiox12040943 PMC10135875 37107317

[11] Kim, K., Park, J., Sohn, Y., Oh, C. E., Park, J. H., Yuk, J. M. & Yeon, J. H. Stability of plant leaf-derived extracellular vesicles according to preservative and storage temperature. *Pharmaceutics*, **14**(2) (2022). 10.3390/pharmaceutics14020457 PMC8879201 35214189

[12] Leng, Y., Yang, L., Zhu, H., Li, D., Pan, S. & Yuan, F. Stability of blueberry extracellular vesicles and their gene regulation effects in intestinal Caco-2 cells. *Biomolecules*, **13**(9) (2023). 10.3390/biom13091412 PMC10526224 37759813

[13] Yuan, H., Yang, M., Han, X. & Ni, X. The Therapeutic Effect of the Chinese Herbal Medicine, Rehmanniae Radix Preparata, in Attention Deficit Hyperactivity Disorder via Reversal of Structural Abnormalities in the Cortex. *Evid. Based Complem. Altern. Med. eCAM* **2018**, 3052058 (2018). 10.1155/2018/3052058 PMC6204205 30405737

[14] Zhang, R. X., Li, M. X. & Jia, Z. P. Rehmannia glutinosa: Review of botany, chemistry and pharmacology. *J. Ethnopharmacol.* **117**(2), 199–214 (2008). 18407446 10.1016/j.jep.2008.02.018

[15] Bian, Z. *et al.* Extraction, structure and bioactivities of polysaccharides from *Rehmannia glutinosa*: A review. *J. Ethnopharmacol.* **305**, 116132 (2023). 36634722 10.1016/j.jep.2022.116132

[16] Huang, Y. *et al.* PEGylated nano-Rehmannia glutinosa polysaccharide induces potent adaptive immunity against Bordetella bronchiseptica. *Int. J. Biol. Macromol.* **168**, 507–517 (2021). 33310103 10.1016/j.ijbiomac.2020.12.044

[17] Qiu, F. S. *et al.* Rgl-exomiR-7972, a novel plant exosomal microRNA derived from fresh Rehmanniae Radix, ameliorated lipopolysaccharide-induced acute lung injury and gut dysbiosis. *Biomed. Pharmacother. Biomed. Pharmacother.* **165**, 115007 (2023). 37327587 10.1016/j.biopha.2023.115007

[18] Gan, R. H. *et al.* Notch1 regulates tongue cancer cells proliferation, apoptosis and invasion. *Cell Cycle (Georgetown, Tex.)* **17**(2), 216–224 (2018). 29117785 10.1080/15384101.2017.1395534 PMC5884382

[19] Zeng, L. *et al.* Aloe derived nanovesicle as a functional carrier for indocyanine green encapsulation and phototherapy. *J. Nanobiotechnol.* **19**(1), 439 (2021). 10.1186/s12951-021-01195-7 PMC8686546 34930289

[20] Kabelka, I. & Vácha, R. Advances in molecular understanding of α-helical membrane-active peptides. *Acc. Chem. Res.* **54**(9), 2196–2204 (2021). 33844916 10.1021/acs.accounts.1c00047

[21] Mu, N. *et al.* Plant-derived exosome-like nanovesicles: Current progress and prospects. *Int. J. Nanomed.* **18**, 4987–5009 (2023). 10.2147/IJN.S420748 PMC10492547 37693885

[22] Ly, N. P., Han, H. S., Kim, M., Park, J. H. & Choi, K. Y. Plant-derived nanovesicles: Current understanding and applications for cancer therapy. *Bioact. Mater.* **22**, 365–383 (2023). 36311046 10.1016/j.bioactmat.2022.10.005 PMC9588993

[23] Fu, C. *et al.* Rehmannioside A improves cognitive impairment and alleviates ferroptosis via activating PI3K/AKT/Nrf2 and SLC7A11/GPX4 signaling pathway after ischemia. *J. Ethnopharmacol.* **289**, 115021 (2022). 35091012 10.1016/j.jep.2022.115021

[24] Xu, L., Zhang, W., Zeng, L. & Jin, J. O. Rehmannia glutinosa polysaccharide induced an anti-cancer effect by activating natural killer cells. *Int. J. Biol. Macromol.* **105**(Pt 1), 680–685 (2017). 28716751 10.1016/j.ijbiomac.2017.07.090

[25] Liu, N. A. *et al.* Rehmannia glutinosa polysaccharide regulates bone marrow microenvironment via HIF-1α/NF-κB signaling pathway in aplastic anemia mice. *Anais da Academia Brasileira de Ciencias* **95**(3), e20220672 (2023). 37556607 10.1590/0001-3765202320220672

[26] Yan, G. *et al.* Brucea javanica derived exosome-like nanovesicles deliver miRNAs for cancer therapy. *J. Control. Release Off. J. Control. Release Soc.* **367**, 425–440 (2024). 10.1016/j.jconrel.2024.01.060 38295998

[27] Sousa de Almeida, M. *et al.* Understanding nanoparticle endocytosis to improve targeting strategies in nanomedicine. *Chem. Soc. Rev.* **50**(9), 5397–5434 (2021). 33666625 10.1039/D0CS01127D PMC8111542

[28] Trenkenschuh, E. *et al.* Enhancing the Stabilization potential of lyophilization for extracellular vesicles. *Adv. Healthc. Mater.* **11**(5), e2100538 (2022). 34310074 10.1002/adhm.202100538 PMC11468620

[29] Yuan, F., Li, Y. M. & Wang, Z. Preserving extracellular vesicles for biomedical applications: Consideration of storage stability before and after isolation. *Drug Deliv.* **28**(1), 1501–1509 (2021). 34259095 10.1080/10717544.2021.1951896 PMC8281093

[30] Frank, J. *et al.* Extracellular vesicles protect glucuronidase model enzymes during freeze-drying. *Sci. Rep.* **8**(1), 12377 (2018). 30120298 10.1038/s41598-018-30786-y PMC6098026

[31] Bosch, S. *et al.* Trehalose prevents aggregation of exosomes and cryodamage. *Sci. Rep.* **6**, 36162 (2016). 27824088 10.1038/srep36162 PMC5099918

[32] Kocholata, M., Prusova, M., Auer Malinska, H., Maly, J. & Janouskova, O. Comparison of two isolation methods of tobacco-derived extracellular vesicles, their characterization and uptake by plant and rat cells. *Sci. Rep.* **12**(1), 19896 (2022). 36400817 10.1038/s41598-022-23961-9 PMC9674704

